# Antigen-Pulsed CpG-ODN-Activated Dendritic Cells Induce Host-Protective Immune Response by Regulating the T Regulatory Cell Functioning in *Leishmania donovani*-Infected Mice: Critical Role of CXCL10

**DOI:** 10.3389/fimmu.2014.00261

**Published:** 2014-06-04

**Authors:** Saikat Majumder, Amrita Bhattacharjee, Bidisha Paul Chowdhury, Suchandra Bhattacharyya Majumdar, Subrata Majumdar

**Affiliations:** ^1^Division of Molecular Medicine, Bose Institute, Kolkata, India

**Keywords:** *Leishmania*, T regulatory cells, vaccine, dendritic cell, CXCL10

## Abstract

Visceral leishmaniasis (VL), caused by *Leishmania donovani*, is a systemic infection of reticulo-endothelial system. There is currently no protective vaccine against VL and chemotherapy is increasingly limited due to appearance of drug resistance to first line drugs such as antimonials and amphotericin B. In the present study, by using a murine model of leishmaniasis we evaluated the function played by soluble leishmanial antigen (SLA)-pulsed CpG-ODN-stimulated dendritic cells (SLA–CpG–DCs) in restricting the intracellular parasitic growth. We establish that a single dose of SLA–CpG–DC vaccination is sufficient in rendering complete protection against *L. donovani* infection. In probing the possible mechanism, we observe that SLA–CpG–DCs vaccination results in the significant decrease in Foxp3^+^GITR^+^CTLA4^+^CD4^+^CD25^+^ regulatory T cells (Treg) cell population in *Leishmania*-infected mice. Vaccination with these antigen-stimulated dendritic cells results in the decrease in the secretion of TGF-β by these Treg cells by possible regulation of the SMAD signaling. Moreover, we demonstrate that a CXC chemokine, IFN-γ-inducible protein 10 (IP-10; CXCL10), has a direct role in the regulation of CD4^+^CD25^+^ Treg cells in SLA–CpG–DC-vaccinated parasitized mice as Treg cells isolated from IP-10-depleted vaccinated mice showed significantly increased TGF-β production and suppressive activity.

## Introduction

Visceral leishmaniasis (VL), a neglected tropical disease is caused by *Leishmania donovani*, a protozoan parasite (1, 2). *Leishmania* promastigotes infect the cells of the reticulo-endothelial system where they multiply (2). Emergence of severe drug resistance against the first line drugs prompts new therapeutic approach (3, 4).

We have previously reported that mice vaccinated with a single dose of soluble leishmanial antigen (SLA)-pulsed DC stimulated with CpG oligodeoxynucleotides or CpG-ODN (SLA–CpG–DCs) were protected against a subsequent leishmanial challenge (5). Stimulation with CpG-ODN, a TLR9 ligand along with SLA activates the dendritic cells and results in the development of *Leishmania* antigen-specific cytotoxic T lymphocytes, which destroys the parasite *in vivo* (5). CpG–ODN also causes the DCs to produce CXCL10, a CXC chemokine, which has previously reported anti-leishmanial properties (6). Besides dendritic cells, the major producers of CXCL10 are monocytes and macrophages (7). Moreover, a strong induction of CXCL10 is observed in *Leishmania* resistant B6 mice, thus linking it with a strong pro-inflammatory Th1 immune response (8). Earlier from our lab, we have also demonstrated that exogenously administered CXCL10 besides regulating the intracellular parasitic load can also regulate the CD4^+^CD25^+^ regulatory T cells (Treg) cells in *Leishmania*-infected mice (9). In the present study, we have investigated the potential role of SLA–CpG–DCs vaccination in the regulation of immunosuppressive CD4^+^CD25^+^ Treg cells in *Leishmania*-infected mice.

Regulatory T cells are a subpopulation of T cells, which suppress immune responses of other cell types (10). Characteristic markers of Tregs are CD25 (10), glucocorticoid-induced tumor necrosis factor receptor (GITR) (11), cytotoxic T lymphocyte antigen 4 (CTLA4) (12) and Foxp3 (13). Tregs have been reported to play a fundamental role in the progression of leishmanial disease predominantly by suppressing Th1 immune responses (14, 15). Tregs secrete high levels of immunosuppressive Th2 cytokines such as IL-10 and TGF-β (10). IL-10, a classical Th2 cytokine is produced by many different cells such as CD4^+^ T cells, Tregs, macrophages, dendritic cells, and even NK cells (16). However, the major source of IL-10 during human VL is CD4^+^CD25^−^ T cells but not CD4^+^CD25^+^ Treg cells (17). CD4^+^CD25^+^ Tregs on the other hand are the major producers of TGF-β during active VL (18). TGF-β, a regulatory cytokine has several down regulatory effects on the host immune system; it inhibits TNF-α and IFN-γ production from activated T cells (19), decreases the nitric oxide production (20) and also abrogates the antigen presenting function of host macrophages (21). Treatment with neutralizing antibodies to TGF-β to susceptible mice results in resistance to *Leishmania* infection (22). Besides, TGF-β is also important for the *in vivo* expansion of CD4^+^CD25^+^ Treg cells (23). Tregs isolated from TGF-β-deficient mice are defective in their suppressive property (24). Effective TGF-β signaling in Tregs also requires phosphorylation and subsequent nuclear translocation of SMAD proteins specifically SMAD4 (25–27).

Our results show that SLA–CpG–DCs vaccination inhibits the generation of CD4^+^CD25^+^ Treg cells in *Leishmania*-infected mice along with the TGF-β production by these Tregs by possible regulation of SMAD signaling. In addition, we demonstrate that the SLA–CpG–DCs-mediated reduction of Tregs can be entirely attributable to CXC chemokine IP-10, as significant increase in CD4^+^CD25^+^ Treg cells is observed in SLA–CpG–DCs-vaccinated parasitized mice depleted of IP-10.

## Materials and Methods

### Ethics statement

This study was carried out in strict accordance with the recommendations in the Guide for the Care and Use of Laboratory Animals of the National Institutes of Health. All experimental animal protocols received prior approval from the Institutional Animal Ethical Committee (Bose Institute, Registration Number: 95/99/CPCSEA).

### Animals, parasites, and reagents

BALB/c mice were purchased from the National Center for Laboratory Animal Sciences, India. For each experiment, 8–10 mice (4–6 weeks old) were used, regardless of sex. *L. donovani* strain AG-83 (MHOM/IN/1983/AG-83) was maintained *in vitro* in Medium 199 (Sigma) containing 10% fetal calf serum (FCS; Gibco BRL). Experiments were performed with stationary phase promastigotes. The CpG-ODN 1826 (5′-TCCATGACGTTCCTGACGTT-3′) and the control-ODN (non-CpG-ODN, 5’-TCCATGAGCTTCCTGAGCTT-3’) was obtained from InvivoGen. CXCL10-depleting antibody was obtained from R&D Systems.

### Preparation of dendritic cells

Bone marrow-derived DCs from BALB/c mice were generated as described previously (28). Non-adherent cells were collected, and 1 × 10^6^ cells were placed in plates containing 1 ml of complete medium with GM-CSF (150 U/ml; R&D Systems) and IL-4 (75 U/ml; R&D Systems) as originally described earlier (5). Half of the medium was replaced on day 3, 5, and 7 and fresh medium containing GM-CSF and IL-4 was added. On day 8 of culture, most cells had acquired typical dendritic morphology. These cells were used as the source of DCs in subsequent experiments.

### DC vaccination

For DC-based vaccination, DCs were pulsed with both SLA and CpG-ODNs (29) as originally described earlier (5). In case of dual stimulation, CpG-ODN (10 μg/ml) or control-ODN (10 μg/ml) was added to the media for last 6 h after 12 h of SLA stimulation. DCs were then washed with PBS thrice and injected i.v. (10^6^ cells in 100 μl of PBS/mouse) into mice through the tail vein. One week later, mice were infected intravenously with 1 × 10^7^ stationary phase *L. donovani* promastigotes. Mice were sacrificed on day 56 post-infections. Spleen and liver parasitic loads were determined from Giemsa-stained impression smears, calculated as the number of parasites per 1000 nucleated cells × organ weight (in milligrams) and expressed in Leishman Donovan Units (LDU) (30). After 28 days of infection, spleens from infected BALB/c mice were removed aseptically, and a single-cell suspension was prepared. Briefly, spleen homogenate was subjected to centrifugation on a Histopaque-1077 (Sigma) gradient and splenocytes were collected, washed, and resuspended in RPMI-1640 complete medium supplemented with 10% FCS.

### *In vivo* depletion of CXCL10

For *in vivo* depletion of CXCL10, anti-mouse CXCL10 mAb (R&D Systems) were injected intraperitoneal (i.p.) on day 0 (250 mg), day 2 (100 mg), and day 4 (100 mg) after SLA–CpG–DCs vaccination as originally described earlier (5). These mice were subsequently infected with 1 × 10^7^ stationary phase *L. donovani* promastigotes after 7 days of initial vaccination. Two hundred fifty milligrams of anti-CXCL10 mAb was again injected i.p on days 10, 15, and 24 of initial vaccination. Depletion efficiencies were assessed at regular intervals.

### Purification of CD4^+^ T cells

CD4^+^ T cells were purified from splenocytes from differently treated mice by positive selection using magnetic beads as originally described earlier (9). CD4^+^ T cells were purified by anti-mouse CD4 (L3T4)-magnetic particles (BD Biosciences). To further separate CD4^+^ T cells into CD25^+^ and CD25^−^ populations, total CD4^+^ T cells were isolated by negative selection using magnetic beads followed by positive selection using anti-CD25 magnetic beads on a magnetic separator column into CD4^+^CD25^+^ and CD4^+^CD25^−^ populations as per manufacturer’s suggested protocol (MagCellect Treg isolation kit, R&D Systems). The cells were stained with anti-CD25 mAb, and the purity of cell preparations was determined by using FACS analysis (FACSCalibur; BD Labware). The purities of CD4^+^CD25^+^ and CD4^+^CD25^−^ T cells were routinely >90 and 99%, respectively.

Additional analyses of T cell phenotypes were also performed using FACS where splenocytes were stained using 1 μg Ab/1 × 10^6^ cells and either run immediately or fixed (3% paraformaldehyde in PBS). In some cases, the splenocytes were first stained with CD4 FITC followed by permeabilization using FACS permeabilizing solution (BD Pharmingen) and FOXP3-PE staining. The panel of Abs used for T cell phenotyping included the following: CD4, CD25, GITR, Foxp3, and CTLA4 (BD Biosciences). The dot plots were derived from the gated events based on the region encircling lymphocytes, which was set using a forward versus side scatter display, and all fluorescent parameters were gated on this population and analyzed on a Flow cytometer (FACSCalibur), using the Cell Quest program on at least 10,000 events.

### Proliferation assay and cytokine ELISA

Splenic responder CD4^+^CD25^−^ T cells (5 × 10^5^) and T-depleted, mitomycin C-treated syngeneic APCs (5 × 10^5^) were cultured in the absence or presence of increasing numbers of splenic CD4^+^CD25^+^ Treg cells for 4 days in 96-well, round-bottom plates as originally described earlier (9). Soluble leishmanial antigen (SLA) (10 μg/ml) was added to the culture for stimulation. One microcurie of [3H] thymidine was added 18 h before harvesting, and incorporated radioactivity was determined on a beta emission reader. Supernatants were collected from the co-culture of responder CD4^+^CD25^−^ and CD4^+^CD25^+^ Treg cells (1:1) at 24 h (for IL-2) or 72 h (for other cytokines). In some cases, splenocytes (2 × 10^6^ cells/ml per well), CD4^+^CD25^−^ T cells (1 × 10^6^ cells/ml per well), or CD4^+^CD25^+^ Treg cells (1 × 10^6^ cells/ml per well) from different sets of treatment were stimulated with SLA (10 μg/ml) for 24 h (IL-2) or 72 h (for other cytokines). The levels of cytokines in supernatants were determined by specific ELISAs using paired mAbs for IL-2, IL-10, IFN-γ, and TGF-β along with the appropriate mouse cytokine controls (BD Biosciences and R&D Systems).

### Flow cytometry

For intracellular cytokine analysis, flow cytometry was performed for the determination of IFN-γ, IL-12, TGF-β, and IL-10 produced by CD4+ T cells in differently treated mice at the single-cell level as originally described earlier (9). In another experiment, CD4^+^CD25^+^ Treg cells were analyzed for Foxp3-positive Treg cells producing TGF-β. Splenocytes or MACS-purified CD4^+^CD25^+^ Treg cells from different groups of experimental mice were stimulated for 20–24 h with SLA (10 μg/ml). Brefeldin A (10 μg/ml) was added to the cultures 2 h before harvesting. The cells were washed in PBS containing 0.1% NaN_3_/1% FCS at 4°C, and some sets were stained with FITC-conjugated anti-CD4. Cells were then permeabilized by treatment with FACS permeabilizing solution (BD Pharmingen) and stained with PE-conjugated IFN-γ, FITC-conjugated IL-12, FITC or PE-conjugated anti-mouse TGF-β mAb, PE-conjugated anti-mouse IL-10 mAb, PE-conjugated Foxp3 anti-hamster mAb, or isotype-matched control mAb and analyzed on a Flow cytometer (FACSCalibur) using the Cell Quest program on at least 10,000 events.

### Real-time PCR quantification

Total RNA was extracted from 2 × 10^6^ CD4^+^CD25^+^ T cells or CD4^+^CD25^−^ T cells with use of TRI Reagent (Sigma), according to the manufacturer’s protocol as originally described previously (9). Isolated RNA (1 μg) was then reverse transcribed using Revert Aid M-MuLV Reverse Transcriptase (Fermentas). The resulting complementary DNA was used for real-time (RT) PCR to detect different Treg cell-specific markers with use of the ABI 7500 RT-PCR system with the DNA-binding SYBR green dye (Applied Biosystems). Glyceraldehyde-3 phosphate dehydrogenase (GAPDH) was used as a reference. The forward- and reverse-specific primer sequences used were as follows: CTLA4 Forward, 5′-GGACGCAGATTTATGTCATTGATC-3′, CTLA4 Reverse, 5′-CCAAGCTAACTGCGAC AAGGA-3′; Foxp3 Forward, 5′-CGTACACCCAGGAAAGACAG-3′, Foxp3 Reverse, 5′-ATCCAGGAGATGATCTGCTTG-3′; GITR Forward, 5′-GACGGTCACTGCAGACTTTG-3′; GITR Reverse, 5′-GCCATGACCAGGAAGATGAC-3′. The reaction conditions involved an initial activation step (5 min at 95°C) and a cycling step (denaturation for 30 s at 94°C, annealing for 30 s at 58.5°C, and extension for 1 min at 72°C for 40 cycles), followed by melting curve analysis. Detection of dequenched probe, calculation of threshold cycles, and further analysis of these data were performed using the Sequence Detector software (Applied Biosystems). Relative changes in CTLA4, Foxp3, and GITR messenger RNA (mRNA) expression were compared with unstimulated control, normalized to GAPDH, and quantified by the 2^−ddCt^ method.

### Preparation of cell lysate and immunoblot analysis

Cell lysates were prepared as described earlier (31). Equal amounts of protein (30 μg) in each lane were subjected to SDS-10% polyacrylamide gel electrophoresis and transferred to a nitrocellulose membrane. The membrane was blocked overnight with 3% BSA in Tris–saline buffer (pH7.5), and immunoblotting was carried out for detecting phosphorylated or dephosphorylated forms of SMAD4 as described previously (32).

### Statistical analysis

A minimum of five mice were used per group for *in vivo* experiments. The data, represented as mean ± standard deviation (SD), is from one experiment, which was performed at least three times. Student’s *t*-test was employed to assess the significance of the differences between the mean values of control and experimental groups. A *P* value of 0.05 was considered significant and <0.001 was considered highly significant.

## Results

### SLA–CpG–DC vaccination mediates effective protection against visceral leishmaniasis through a potent pro-inflammatory response

**Figure 1:**
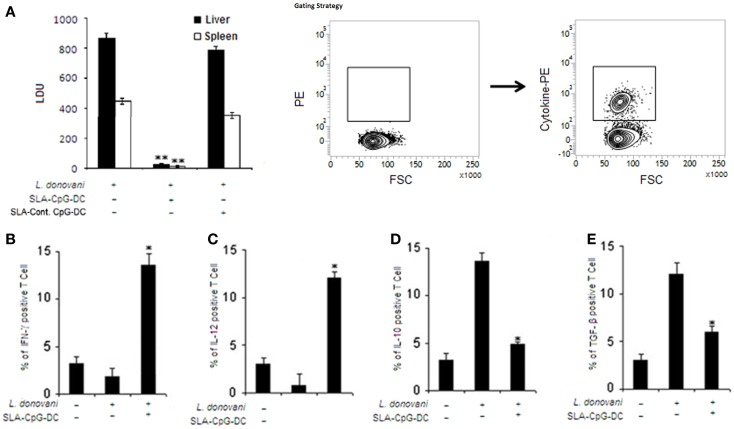
**Soluble leishmanial antigen–CpG–DC vaccination mediates effective protection against visceral leishmaniasis through a potent pro-inflammatory response**. **(A)** Mice were vaccinated with SLA and CpG-ODN-pulsed DCs, SLA and control-ODN-pulsed DCs, or phosphate buffered saline (PBS; control) followed by intravenous infection with 1 × 10^7^ stationary phase *Leishmania donovani* promastigotes after 7 days. Mice were sacrificed on day 56 after infection. Levels of parasite burden in liver and spleen samples were determined by stamp–smear method and expressed in Leishman Donovan Units (LDU). Results are from three independent experiments and represent the mean values ± standard errors of the means for five animals per group per time point. **P < 0.001, compared to PBS-treated infected mice. In another set of experiments, splenocytes (2 × 10^6^) from control, *L. donovani*-infected (28 days), and SLA–CpG–DC-vaccinated infected mice (28 days) were assessed for intracellular **(B)** IFN-γ, **(C)** IL-12, **(D)** IL-10, or **(E)** TGF-β staining, which was performed as mentioned in Section “Materials and Methods” and analyzed by flow cytometry. Magnetically purified CD4^+^ T cells were analyzed for IL-12-PE, IFN-γ-PE, IL-10-PE, or TGF-β-PE staining to detect CD4^+^IL-12^+^, CD4^+^ IFN-γ^+^, CD4^+^IL-10^+^, or CD4^+^ TGF-β^+^ T cells. The bar graphs represent the mean dot plot values based on the region encircling positive cells from three independent experiments *P < 0.05, compared with infected sets. The error bars represent mean ± SD of three mice per group.

Our results demonstrated that SLA-pulsed DCs in the presence of TLR9 agonist, CpG-ODN (SLA–CpG–DCs), acquired the ability to induce complete protection against *L. donovani*. SLA–CpG–DCs-vaccinated BALB/c mice on day 56 of infection, showed marked decrease in parasitic burden; 96 ± 2.6 and 97 ± 1.9% reduction in hepatic and splenic parasite burden, respectively, compared with PBS-treated infected controls (Figure 1A). Mice vaccinated with SLA and control-ODN-stimulated dendritic cells (SLA-Cont. ODN–DCs) were unable to give any protection against the leishmanial challenge (Figure 1A). Moreover, the protection conferred by SLA–CpG–DC vaccination against *L. donovani* infection is significantly dependent on a Th1 polarized anti-parasitic immune response (Figures 1B,C). Splenocytes from differently vaccinated mice at 28 days post-treatment were re-stimulated with SLA to evaluate the percentages of T cells secreting various pro-inflammatory or anti-inflammatory cytokines. There was about sixfold increase in IFN-γ secreting CD4^+^ T cells along with nearly fivefold increase in IL-12 secreting CD4^+^ T cells in splenocytes from SLA–CpG–DC-vaccinated parasitized mice compared with only infected mice (Figures 1B,C). Moreover, there was nearly fourfold decrease in TGF-β secreting CD4^+^ T cells along with nearly threefold decrease in IL-10-secreting CD4^+^ T cells in splenocytes from SLA–CpG–DC-vaccinated parasitized mice compared with only infected mice (Figures 1D,E).

### SLA–CpG–DCs vaccination leads to decrease in splenic CD25^+^CTLA4^+^GITR^+^Foxp3^+^CD4^+^ T cells in *L. donovani*-infected mice

**Figure 2:**
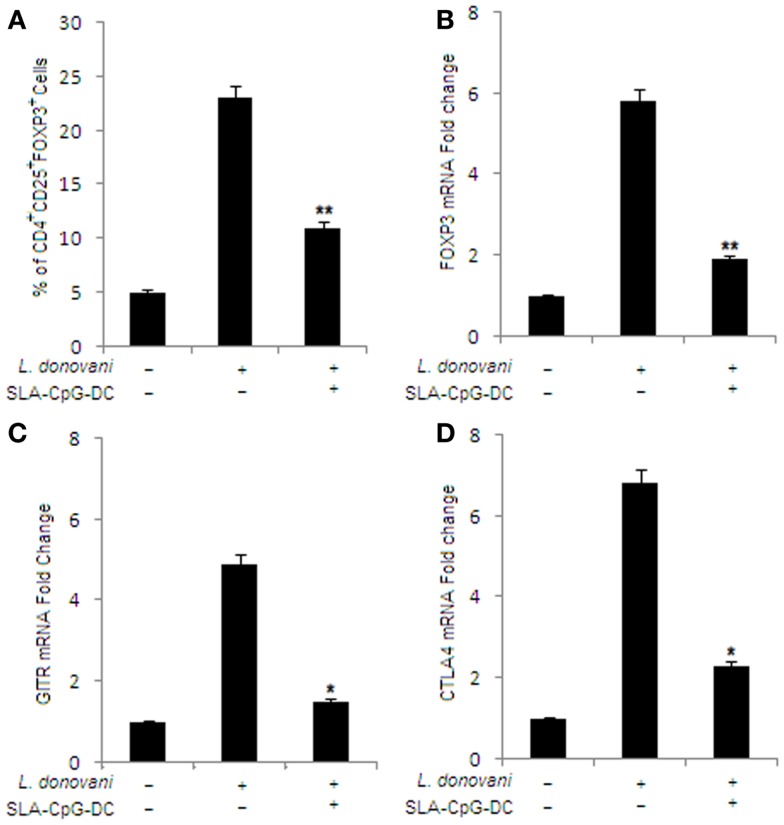
**Effect of SLA–CpG–DCs vaccination on the frequency and phenotype of T regulatory cells**. **(A)** CD4^+^ T cells (1 × 10^6^) were purified from infected and indicated treatment groups of mice 28 days after infection, plated aseptically followed by fixation and staining for T regulatory cell-specific markers like FITC-conjugated CD25 and PE-conjugated FoxP3 mentioned in Section “Materials and Methods.” The data was analyzed by flow cytometry in each group of untreated and differently treated BALB/c mice have been presented. Data represent the mean ± SD for three animals per group. In a separate experiment, CD4^+^CD25^+^ Treg cells (2 × 10^6^) were purified from spleen of differently treated mice by MACS as described in Section “Materials and Methods.” These magnetically purified Treg cells were collected in TRIZOL for mRNA extraction and real-time PCR to study mRNA expression of T regulatory cell markers. Quantitative RT-PCR showing the expression of Foxp3 **(B)**, GITR **(C)**, and CTLA4 mRNA **(D)**, where the data were presented as changes (*n*-fold) from uninfected control cells. *P < 0.05 and **P < 0.001, compared with T regulatory cells isolated from infected mice. The data represent the mean ± SD of data from three independent experiments, which yielded similar results.

To check whether SLA–CpG–DCs vaccination of *L. donovani*-infected mice can modulate the Treg cells, which have been previously reported to play a crucial role in disease progression (33), FACS analysis was performed to evaluate the frequency and phenotype of splenic Treg cell population. We observed a significant decrease from 23% to 11% in CD4^+^CD25^+^Foxp3^+^ Treg cells in splenocytes isolated from SLA–CpG–DCs-vaccinated infected mice compared to only infected mice (Figures 2A,B). Other Treg cell-specific markers like CTLA4 and GITR showed a significant decrease in splenocytes isolated from SLA–CpG–DC-vaccinated infected mice compared to infected sets (Figures 2C,D). We observed 2.5-fold decrease in GITR and threefold decrease in CTLA4 mRNA levels in SLA–CpG–DC-vaccinated infected sets in comparison with only infected sets.

### SLA–CpG–DC vaccination reduces the suppressive properties of the CD4^+^CD25^+^Foxp3^+^ T cells in *L. donovani*-infected mice

**Figure 3:**
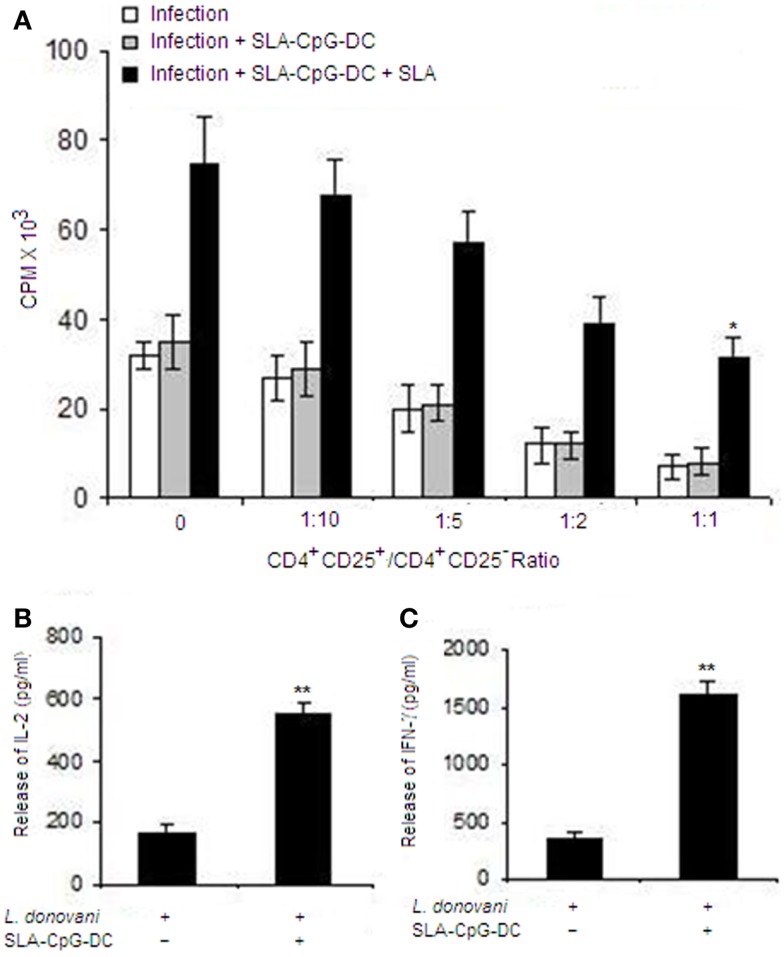
**Soluble leishmanial antigen–CpG–DCs vaccination reduces the suppressive properties of the CD4^+^CD25^+^Foxp3^+^ T cells in *L. donovani*-infected mice**. **(A)** CD4^+^CD25^+^ T regulatory cells and CD4^+^CD25^−^ T cells were purified from spleen of differently treated mice (Materials and Methods) after 4 weeks post-infection. CD4^+^CD25^−^ T cells (5 × 10^5^) and T-depleted, mitomycin C-treated syngeneic APCs (5 × 10^5^) were stimulated with SLA (10 μg/ml) and co-cultured in the presence of splenic CD4^+^CD25^+^ T cells(1:1 ratio) from differently treated sets for 4 days. Proliferation was determined by an 18 h (^3^H) thymidine incorporation assay. Data are presented as counts per minute (CPM) × 10^3^. The supernatants were collected at 24 h for IL-2 **(B)** or at 72 h for IFN-γ **(C)** following stimulation with SLA (10 μg/ml) and levels of cytokines were determined by ELISA. The error bars represent mean ± SD of triplicate cultures. The experiments were performed three times, of which one set of representative data is shown (**P < 0.001 and *P < 0.05).

To study whether the Treg cells isolated from the *Leishmania*-infected BALB/c mice can suppress the function of effector T cells, we performed co-culture experiments using CD4^+^CD25^+^ and CD4^+^CD25^−^ T cells purified from spleen of differently treated mice. We observed that CD4^+^CD25^+^ Treg cells isolated from untreated infected mice can efficiently suppress the proliferation of responder CD4^+^CD25^−^ T cells in a dose-dependent manner. Whereas, CD4^+^CD25^+^ Treg cells, isolated from SLA–CpG–DC-vaccinated infected mice, could not suppress the proliferation of responder CD4^+^CD25^−^ T cells (Figure 3A).

Moreover, CD4^+^CD25^+^ Treg cells from SLA–CpG–DC-vaccinated mice failed to suppress the release of IL-2 when co-cultured with CD4^+^CD25^−^ responder T cells (1:1) compared to CD4^+^CD25^+^ Treg cells isolated from infected mice (Figure 3B). Additionally, CD4^+^CD25^+^ Treg cells from infected mice significantly abrogated the IFN-γ secretion by responder CD4^+^CD25^−^ T cells, while CD4^+^CD25^+^ Treg cells from SLA–CpG–DC-vaccinated parasitized mice, could not suppress the IFN-γ production from responder CD4^+^CD25^−^ T cells demonstrating parasite-specific T cell responses (Figure 3C).

### SLA–CpG–DC vaccination leads to reduced TGF-β secretion from T regulatory cells in *L. donovani*-infected mice

**Figure 4:**
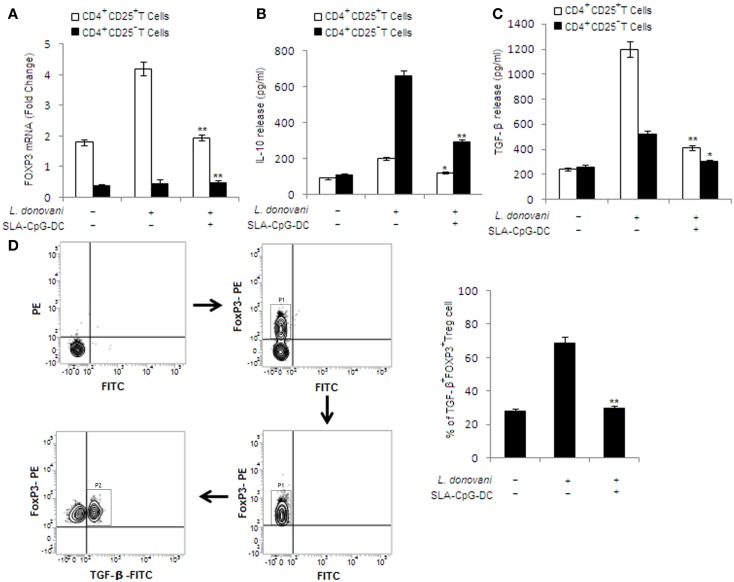
**Effect of SLA–CpG–DC vaccination on IL-10 and TGF-β release from T regulatory cells**. **(A)** CD4^+^CD25^+^ Treg cells or CD4^+^CD25^−^ T cells (2 × 10^5^), purified from spleen of differently treated mice (see Materials and Methods) 4 weeks post-infection, were collected in TRIZOL for mRNA extraction and real-time PCR to study Foxp3 mRNA expression or were stimulated with SLA for 72 h, after which the cell supernatants were collected for estimation of IL-10 **(B)** and TGF-β **(C)** by ELISA. All data were presented as mean ± SD of three triplicate wells. One of the three independent experiments is shown. *P < 0.001 indicates statistically significant differences compared with infected sets. In a separate experiment, CD4^+^CD25^+^ T regulatory cells (1 × 10^6^), isolated from spleen of differently treated mice (4 weeks post-infection), were assessed for TGF-β and Foxp3 **(D)**, which was performed as mentioned in Section “Materials and Methods,” and analyzed by Flow cytometry. Magnetically purified CD4^+^CD25^+^ cells were analyzed for FoxP3-PE staining to detect FoxP3^+^CD4^+^CD25^+^ cells (P1 gated cell population in the sorting scheme). These FoxP3^+^CD4^+^CD25^+^ cells were further analyzed for TGF-β-FITC staining to detect TGF-β^+^ FoxP3^+^CD4^+^CD25^+^T cells (P2 gated cell population in the sorting scheme). The bar graphs represent the mean dot plot values based on the region encircling positive cells from three independent experiments. **P < 0.001 and *P < 0.05, compared with T regulatory cells isolated from infected mice. The data represent the mean ± SD of data from three independent experiments, which yielded similar results.

SLA–CpG–DC vaccination of *L. donovani*-infected mice results in a significant decrease in Th2 cytokine secreting CD4^+^ T cells, i.e., IL-10 and TGF-β (Figures 4B,C). As expected, FoxP3 levels were higher in CD4^+^CD25^+^ Treg cells isolated from infected group of mice. On the contrary, CD4^+^CD25^−^ T cells showed lower expression of Foxp3 mRNA during infection (Figure 4A). Now to delineate which of these two cells are the major producer of IL-10 and TGF-β, we evaluated the anti-inflammatory cytokines secreted by CD4^+^CD25^+^ Treg cells and CD4^+^CD25^−^ T cells from different groups of mice. CD4^+^CD25^−^ and CD4^+^CD25^+^ T cells from control mice showed lower levels of IL-10 and TGF-β (Figures 4B,C).

In infected mice, CD4^+^CD25^−^ T cells are the major producers of IL-10. On the other hand, CD4^+^CD25^+^ Treg cells are the major source of TGF-β release. Interestingly, significant decrease in TGF-β release from CD4^+^CD25^+^ Treg cells was observed in CD4^+^CD25^+^ Treg cells isolated from SLA–CpG–DC-vaccinated infected sets compared with infected mice, while CD4^+^CD25^−^ T cells showed a significant reduction in IL-10 release in vaccinated group of mice compared with infected sets (Figures 4B,C).

In another experiment, CD4^+^CD25^+^ Treg cells, isolated from different groups of animals, were analyzed for TGF-β secreting Foxp3^+^ Treg cells. We observed higher levels (69.24%) of TGF-β-secreting Foxp3^+^ Treg cells in infected mice, which were significantly abridged (24.56%) in SLA–CpG–DC-vaccinated infected mice (Figure 4D), indicating decrease of TGF-β secretion from CD4^+^CD25^+^Foxp3^+^ Treg cells following SLA–CpG–DC vaccination during VL.

### IP-10 depletion abrogates the TGF-β signaling in CD4^+^CD25^+^ Treg cells in SLA–CpG–DC-vaccinated parasitized mice

**Figure 5:**
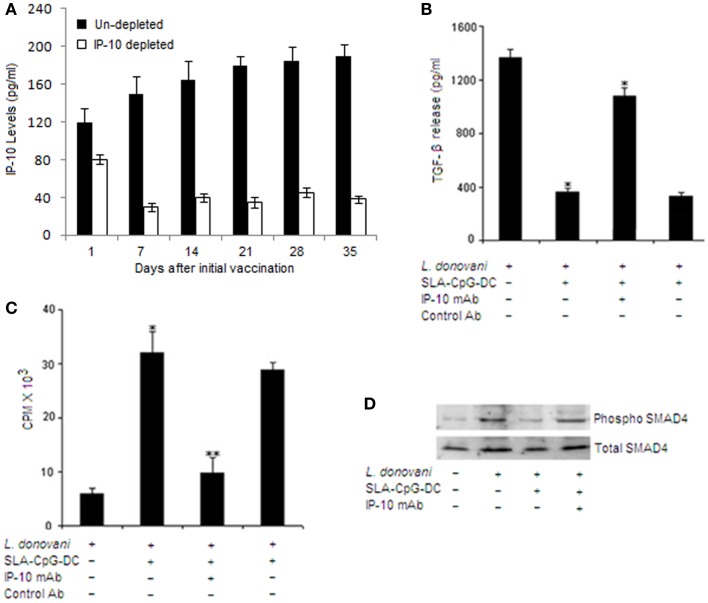
**IFN-γ-inducible protein 10 depletion abrogates the TGF-β signaling in CD4^+^CD25^+^ Treg cells in SLA–CpG–DC-vaccinated parasitized mice**. **(A)** Assessment of depletion efficiency. Levels of IP-10 were measured at indicated days from IP-10 depleted and non-depleted SLA–CpG–DC-vaccinated parasitized mice. Splenic cells were isolated at indicated time points and levels of IP-10 were measured by sandwich ELISA. The experiments were carried out twice. **(B)** CD4^+^CD25^+^ Treg cells (1 × 10^6^) purified from spleen of indicated groups of *L. donovani*-infected (28 days) mice were stimulated with SLA for 72 h. Level of TGF-β in cell culture supernatants of indicated treatment groups was determined by ELISA. Asterisks indicate statistically significant induction (*P < 0.05, **P < 0.001) of TGF-β production compared with infected sets. **(C)** CD4^+^CD25^+^ T regulatory cells and CD4^+^CD25^−^ T cells were purified from spleen of differently treated mice (see Materials and Methods) after 28 days post-infection. CD4^+^CD25^−^ T cells (5 × 10^5^) and T-depleted, mitomycin C-treated syngeneic APCs (5 × 10^5^) were stimulated with SLA (10 μg/ml) in the presence of splenic CD4^+^CD25^+^ T cells (1:1) for 4 days. Proliferation was determined by an 18-h (3H) thymidine incorporation assay. Data were presented as cpm × 10^3^. **P < 0.001 compared with infected sets. **(D)** In a separate experiment, CD4^+^CD25^+^ Treg cells (2 × 10^6^), purified from spleen of indicated groups of mice, were stimulated with SLA for 30 and 60 min. The cells were lysed and subjected to Western blotting with anti-pSMAD4 and anti-SMAD4 as described in Section “Materials and Methods”. The experiments were carried out three times, of which one set of representative data is shown.

We have previously demonstrated that the induction of anti-leishmanial protective immunity by SLA–CpG–DCs is entirely dependent on IP-10 (5). Moreover, our lab has also shown that exogenously administered IP-10 can decrease the TGF-β secretion in *Leishmania*-infected mice (9). So to examine possible involvement of IP-10 in the secretion of TGF-β from CD4^+^CD25^+^ Treg cells following SLA–CpG–DCs vaccination, we vaccinated the mice, infected it with *Leishmania* and monitored the course of infection in the presence or absence of IP-10 (Figure 5A).

CD4^+^CD25^+^ Treg cells from IP-10 depleted SLA–CpG–DC-vaccinated mice produced significantly higher amount of TGF-β together with fourfold increase in their suppressive property compared with IP-10 non-depleted vaccinated parasitized mice, which showed significant reduction in TGF-β secretion along with clear decrease in the suppressive property of the CD4^+^CD25^+^ Treg cells (Figures 5B,C). These findings clearly demonstrate the involvement of IP-10 in the regulation of CD4^+^CD25^+^ Treg cell functioning in SLA–CpG–DC-vaccinated parasitized mice.

Efficient TGF-β signaling requires proper activation of SMAD4 (27). To establish whether SMAD4 is involved in the SLA–CpG–DC-mediated regulation of TGF-β signaling in CD4^+^CD25^+^ Treg cells, we studied the phosphorylation of SMAD4 in CD4^+^CD25^+^ Treg cells during SLA–CpG–DC vaccination. Increased levels of phospho-SMAD4 was observed in CD4^+^CD25^+^ Treg cells isolated from infected mice, however CD4^+^CD25^+^ Treg cells isolated from SLA–CpG–DC-vaccinated mice showed lower levels of phospho-SMAD4 indicating SLA–CpG–DC-mediated regulation of TGF-β signaling through SMAD4 modulation (Figure 5D). Though, increased levels of phospho-SMAD4 in CD4^+^CD25^+^ Treg cells in IP-10-depleted vaccinated parasitized mice suggest the involvement of IP-10 in the SLA–CpG–DCs-mediated suppression of TGF-β.

## Discussion

Our results describe a novel strategy using a DC-based vaccination to confer significant protection against *Leishmania* pathogen. SLA–CpG–DC vaccination of *L. donovani*-infected mice showed significant protection as marked reduction in the hepatic and splenic parasitic burden is observed (Figure 1A). Besides this, significantly higher numbers of IL-12 and IFN-γ secreting T cells are observed in SLA–CpG–DC-vaccinated parasitized mice compared to unvaccinated parasitized mice. At the same time lower numbers of TGF-β and IL-10-producing CD4^+^ T cells in vaccinated mice indicate efficient suppression of Th2 cytokines (Figures 1B–E). Besides CD4^+^ T cells, SLA–CpG–DC vaccination also reduces the number of CD25^+^Foxp3^+^CD4^+^ T regulatory cells in the parasitized mice along with their immunosuppressive molecules GITR and CTLA4 (Figures 2A–D).

Recently it has been shown that CD4^+^CD25^−^ T cells derived, but not CD4^+^CD25^+^ derived IL-10 plays a major role in the murine model of VL (34, 35). Our results also indicate that SLA–CpG–DC vaccination abrogates the secretion of IL-10 from CD4^+^CD25^−^ T cells, which can be crucial for the parasite clearance (Figure 4B). CD4^+^CD25^+^ Treg cells from infected mice, on the other hand secrete higher amounts of TGF-β (Figure 4C). TGF-β, while inhibiting pro-inflammatory cytokine responses aids in the multiplication of the parasite inside the host (36). Additionally, TGF-β is also critical for the effective functioning of Treg cells and has been implicated to play a critical role in the persistence of *Leishmania* infection. Vaccination with SLA–CpG–DC inhibits TGF-β production from CD4^+^CD25^+^Foxp3^+^ Treg cells (Figure 4D) and also reduces their suppressive activity (Figure 3A), which is critical for the resistance against *Leishmania* (37).

Besides, TGF-β plays a decisive role in the generation and expansion of Treg cells and also in the induction of FoxP3 and CTLA4 in Treg cells (27, 38). SMAD proteins play a critical role here for the efficient TGF-β signaling (27). Our results indicate that SLA–CpG–DC vaccination restricted the parasite-induced enhanced phosphorylation of SMAD4 (Figure 5D) and thus can efficiently regulate the TGF-β signaling in Treg cells. The use of neutralizing IP-10 antibody during SLA–CpG–DC vaccination provided evidence that IP-10 besides regulating TGF-β signaling, also regulates the suppressive activity of these Treg cells (Figures 5B,C). Our previous finding revealed that SLA–CpG–DCs vaccination results in significant enhancement of IFN-γ secreting CD8^+^ cytotoxic T lymphocytes along with significant increase in granzyme and perforin secreting CD8^+^ T cells, which most likely contributes to the protection (5). The evidence presented here indicates that the protection was also dependent on the reduction of CD4^+^CD25^+^Foxp3^+^ Treg cells. Collectively, these findings illustrate that SLA–CpG–DCs vaccination induces a strong Th1 response by effective modulation of Treg cell functioning and activation of cytotoxic CD8^+^ T cells, representing an antigen-specific immune response against *Leishmania*-induced pathogenesis.

## Conflict of Interest Statement

The authors declare that the research was conducted in the absence of any commercial or financial relationships that could be construed as a potential conflict of interest.
